# Cytokeratin on Frozen Sections of Sentinel Node May Spare Breast Cancer Patients Secondary Axillary Surgery

**DOI:** 10.1155/2012/802184

**Published:** 2012-05-09

**Authors:** Elisabeth Specht Stovgaard, Tove Filtenborg Tvedskov, Anne Vibeke Lænkholm, Eva Balslev

**Affiliations:** ^1^Department of Pathology, Herlev Hospital, 2730 Herlev, Copenhagen, Denmark; ^2^Department of Breast Surgery, Rigshospitalet, 2100 Copenhagen, Denmark; ^3^Department of Pathology, Rigshospitalet, 2100 Copenhagen, Denmark; ^4^Department of Pathology, University Hospital of Copenhagen, 2100 Copenhagen, Denmark

## Abstract

*Background*. The feasibility and accuracy of immunohistochemistry (IHC) on frozen sections, when assessing sentinel node (SN) status intraoperatively in breast cancer, is a matter of continuing discussion. In this study, we compared a center using IHC on frozen section with a center not using this method with focus on intraoperative diagnostic values. *Material and Methods*. Results from 336 patients from the centre using IHC intraoperatively were compared with 343 patients from the center not using IHC on frozen section. Final evaluation on paraffin sections with haematoxylin-eosin (HE) staining supplemented with cytokeratin staining was used as gold standard. *Results*. Significantly more SN with isolated tumor cells (ITCs) and micrometastases (MICs) were found intraoperatively when using IHC on frozen sections. There was no significant difference in the number of macrometastases (MACs) found intraoperatively. IHC increased the sensitivity, the negative predictive value, and the accuracy of the intraoperative evaluation of SN without decreasing the specificity and positive predictive value of SN evaluation. *Conclusions*. IHC on frozen section leads to the detection of more ITC and MIC intraoperatively. As axillary lymph node dissection (ALND) is performed routinely in some countries when ITC and MIC are found in the SN, IHC on frozen section provides valuable information that can lead to fewer secondary ALNDs.

## 1. Introduction

Axillary lymph node status is the most important prognostic factor in breast cancer. Sentinel node biopsy (SNB) is regarded as an accurate method for assessing axillary lymph node status in patients with early breast cancer [1, 2]. The more comprehensive analysis of SNs has resulted in increased detection of metastatic and micrometastatic disease both by haematoxylin-eosin (HE) staining and by immunohistochemistry (IHC) [3–5]. Only in case of involvement of the SN are patients offered an axillary lymph node dissection (ALND). If SN involvement is found intraoperatively, ALND can be done immediately at the same operation and patients are spared a two-stage procedure. However, there is no universal agreement on the optimal method for the intraoperative examination of the SNs. A higher number of SN involvement diagnosed correctly intraoperatively would increase the number of patients having ALND in the same operation and fewer would go through a second axillary operation because of late diagnosis of SN involvement.

In 2003, the American Joint Committee on Cancer (AJCC) revised the breast cancer staging system (6th edition) with particular attention to the distinction of micrometastases (MICs) and isolated tumor cells (ITCs) [6]. This revision classifies ITC (≤0.2 mm) as node-negative (pN0(i+)). ITCs are defined as clusters of single tumor cells which are usually detected only by IHC or molecular methods but can be verified on HE-stained sections. Cases with MIC (>0.2 mm to 2 mm) are now classified as node-positive (pN1mi). These recent changes to the AJCC staging for lymph nodes containing ITC and MIC have standardized the classification of these metastases. In Denmark, ALND is at the moment routinely performed on patients with ITCs and MICs as well as macrometastases (MACs). Internationally, however, there are diverging opinions as to the necessity of ALND for ITC and MIC and this is not standard procedure in all countries.

The aim of this study was to investigate the feasibility and accuracy of IHC staining in detecting axillary node involvement intraoperatively. We determined the concordance between intraoperative frozen section evaluations with and without IHC and final standard evaluation on HE-stained paraffin sections supplemented with cytokeratin staining with regard to the detection of MAC, MIC, and ITC.

## 2. Material and Methods

SNs from 336 breast cancer patients operated at Herlev Hospital during the period January 1, 2009 to July 14, 2009were evaluated intraoperatively with anticytokeratin IHC on frozen sections (group 1). Sentinel nodes removed were sent for frozen section. Lymph nodes measuring more than 4 mm in diameter were bisected, and very large lymph nodes were sliced at 2-3 mm intervals. Frozen step sections were done at two levels 200 microns apart with 2 HE and one section with anticytokeratin AE1/AE3 IHC (DAKO, ready to use). This is a quick method with a turnaround time of 20 minutes resulting in only minor prolongation of surgery [7]. Intraoperative analysis was limited to positive (any involvement) versus negative, and no attempt to subclassify positives into MAC/MIC/ITC was done.

343 patients operated at Rigshospitalet during the same period were evaluated intraoperatively using the same method as described for group 1, but without cytokeratin staining (group 2). In both groups, the sentinel nodes were formalin fixed and paraffin sections (2 levels were cut, 500 microns apart) were done with HE staining supplemented with cytokeratin staining.

The nodal involvement was classified according to the 6th edition of the AJCC manual. Consistent with standard clinical practice, the pathology is reported on a patient basis, based on the most extensive disease found in any of the SNs. Patients listed on the pathology report as having less than 10 tumor cells identified (according to DBCG guidelines) or nodal involvement ≤0.2 mm on HE or IHC were classified as ITC. If the pathology report did not specify the size or did not specifically report ITCs, the patient was classified in the MAC group. The total number of SNs removed and the number of sentinel nodes with MAC, MIC, ITC, and no involvement were registered for each individual patient both with regard to the frozen section evaluation and with regard to the final evaluation.

Comparisons of results between group 1 and 2 were made by the chi-square test except in cases where the number of observations was less than 40, in which case Fisher's exact test was used.

## 3. Results

SN status for group 1 is shown in Table 1 and, for group 2, in Table 2. In group 1, one patient was excluded as IHC on frozen section failed. In group 2, 7 patients were excluded due to lack of a Danish registration number and therefore no possibility of follow-up.

The total number of patients with nodal involvement (MAC, MIC, and ITC) at the final evaluation on paraffin sections was 118 out of 335 in group 1 and 116 out of 336 in group 2. There was no significant difference between the two groups with regard to frequency of SN involvement on final evaluation (*P* = 0.88).

In group 1, 103 out of 118 patients had nodal involvement correctly diagnosed on frozen sections. This was significantly better than in group 2 where 70 out of 116 patients had nodal involvement correctly diagnosed on frozen sections (*P* < 0.02).

Diagnostic values for frozen section with and without IHC are presented in Table 3 and for MIC/ITC versus MAC with and without IHC in Table 4.

We found a statistically significant difference between the number of MIC found on frozen sections with IHC and without IHC (*P* < 0.005). Furthermore, we found a statistically significant difference between the number of ITC found on frozen sections with IHC and without IHC (*P* = 0.001).

On the contrary, the number of MAC found on frozen sections with IHC was not significantly different from the number of MAC found without IHC (*P* = 0.88).

222 patients with MAC, MIC, or ITC had ALND. 12 patients did not have ALND despite having involvement in the SN due to comorbidity or the patient's wishes. For 8 of these patients, the nodal involvement was not found until final evaluation, while, in 4 patients, involvement was found on frozen section. These 4 patients had minimal involvement of the SNs, that is, ITC or MIC, and didnot therefore have ALND due to comorbidity of the patients.

## 4. Discussion

In the present study, we compared results from a center using only frozen section intraoperatively with results from a center using frozen section with IHC. We found a significantly higher number of MIC and ITC correctly diagnosed intraoperatively when IHC was applied than without. The number of MAC was not significantly different. This is in accordance with other studies on intraoperative IHC, where the difference in positive findings was also seen for MIC and ITC. The ability for frozen section to diagnose MAC, however, seems to be as good as frozen section with IHC. This is further underlined by the diagnostic values shown in Table 4.

For frozen section without IHC, previous studies have found sensitivities of 36–95.8% [8–12], accuracies of 83–96% [8, 9, 12, 13], and negative predictive values of 81–98% [9, 10, 14]. Corresponding values for frozen section with IHC and IHC alone have been 83–92% (sensitivity) [13–16], 92.2–97.5% (accuracy) [11, 13, 15], and 86–95.2% (negative predictive value) [13, 14]. Specificities and positive predictive values are usually very high for both methods. Our results fall within the range of these previous findings.

The large divergence in results seen in other studies may be due to differences in techniques and number of sections both intraoperatively and at postoperative examination. Also, many of the studies have included relatively few patients. In contrast, our study is based on a relatively large number of patients. We show that IHC intraoperatively can be a valuable addition to frozen section, using a method that is feasible in the clinic, that is, not too time consuming. However, it must be taken into consideration that this was a retrospective study and that the patients who had frozen sections without IHC came from a different, although similar, center than the ones who had frozen sections with IHC. Hence, it is possible that differences in technique between the two centers may have influenced the results.

A correct diagnosis of SN status intraoperatively can reduce the number of patients offered ALND as a second operation. When intraoperative IHC was first introduced at our hospital, a study was undertaken to evaluate the economic implications of a slightly prolonged first surgery compared to fewer patients having to go through a later ALND. The cost analysis at that time showed a cost saving of approximately 40% [7]. Another study has shown an increased length of hospitalization and operating time for patients who had late ALND [17]. Few studies have investigated objective and subjective arm morbidity in patients undergoing late ALND compared to those having only one operation, and results are diverging [17–20]. Still, immediate staging of the axilla by SNB and subsequent ALND if necessary under the same operation must be preferred.

8 of the 12 patients in our study who did not have ALND despite involvement in the SN had their nodal involvement diagnosed on final evaluation. Patients having to undergo a second operation for ALND may be more prone to declining ALND to avoid a second hospitalization. However, this study did not provide data as to why the patients declined ALND.

However, the benefit of discovering ITC and MIC intraoperatively depends on how patients with these findings are managed further. The optimal treatment strategy for these patients is now being discussed, and it is questioned whether there is any prognostic advantage in performing ALND. Recently published data showed no survival benefit from ALND in patients with limited SN metastatic breast cancer treated with breast conservation and systemic therapy [21]. If the future management of breast cancer with ITC or MIC in the sentinel node does not involve ALND, there will be no added benefit from the detection of metastases intraoperatively compared to final evaluation.

## 5. Conclusion

IHC intraoperatively adds information to frozen section evaluation. In the present study, IHC increased the sensitivity, the negative predictive value, and the accuracy of the intraoperative evaluation of SN without decreasing the specificity and positive predictive value, of SN evaluation. However, intraoperative IHC only increased the number of patients diagnosed with MIC and ITC, whereas the number of patients with MAC was the same in both groups.

The present study indicates that frozen section with IHC is a clinically useful method as it enables the detection of more nodal involvement intraoperatively, thus allowing ALND to be performed immediately during the same operation in a larger proportion of patients. Hence, a number of patients are spared a second operation. Evidently, however, this method is only relevant as long as patients with ITC and MIC are routinely treated with subsequent ANLD, as is the case in Denmark.

Recently, molecular assays (one-step nucleic acid amplification (OSNA), quantitative reverse transcription-polymerase chain reaction (QRT-PCR)) have been introduced to the intraoperative evaluation of SNs. These procedures yield diagnostic values comparable to those found in our study for IHC on frozen sections [22, 23]. IHC on frozen sections, however, does not require additional equipment and might therefore be an affordable alternative to these molecular assays.

## Figures and Tables

**Table 1:** Comparison of evaluations on frozen sections and paraffin sections of 335 patients with sentinel node biopsies with IHC on frozen sections (group 1).

Final evaluation on paraffin sections	Evaluation on frozen sections		
	No metastasis	Metastasis	Total
No metastasis	217 (100%)	0 (0%)	217
ITC	3 (23%)	10 (77%)	13
MIC	7 (28%)	18 (72%)	25
MAC	5 (6%)	75 (94%)	80

Total	232 (69%)	103 (31%)	335

**Table 2:** Comparison of evaluations on frozen sections and paraffin section of 336 patients with sentinel node biopsies without IHC on frozen sections (group 2).

Final evaluation on paraffin sections	Evaluation on frozen sections		
	No metastasis	Metastasis	Total
No metastasis	220 (100%)	0 (0%)	220
ITC	11 (92%)	1 (8%)	12
MIC	29 (83%)	6 (17%)	35
MAC	6 (9%)	63 (91%)	69

Total	266 (79%)	70 (21%)	336

**Table 3:** Diagnostic values for frozen section with and without IHC.

	IHC	No IHC
Sensitivity	87.3% (103/118)	60.3% (70/116)
Specificity	100% (217/217)	100% (220/220)
Positive predictive value	100% (103/103)	100% (70/70)
Negative predictive value	93.5% (217/232)	82.6% (220/266)
Accuracy	95.5% (320/335)	86.0% (290/336)

**Table 4:** Diagnostic values for MIC/ITC versus MAC.

	IHC		No IHC	
	MIC/ITC	MAC	MIC/ITC	MAC
Sensitivity	73.7% (28/38)	93.8% (75/80)	14.9% (7/47)	91.3% (63/69)
Specificity	100% (217/217)	100% (217/217)	100% (220/220)	100% (220/220)
Positive predictive value	100% (28/28)	100% (75/75)	100% (7/7)	100% (63/63)
Negative predictive value	95.6% (217/227)	97.8% (217/222)	84.6% (220/260)	97.3% (220/226)
Accuracy	96.1% (245/255)	98.3% (292/297)	85% (227/267)	97.9% (283/289)
